# Geriatric Depression Mimicking Parkinson's Disease: A Case of Levodopa‐Responsive Pseudoparkinsonism With a 3‐Year Follow‐Up

**DOI:** 10.1002/ccr3.72682

**Published:** 2026-05-10

**Authors:** Kenshiro Imaizumi, Junichi Goto, Kazuya Isoda, Tetsuya Watanabe, Yohei Nakamura, Suzuha Oyamada, Masashi Toda, Koki Hirazumi, Sachi Yamashiro, Yoshihiro Nabeshima

**Affiliations:** ^1^ Yuge Neuropsychiatric Hospital Kumamoto Japan

**Keywords:** dopaminergic imaging, geriatric psychiatry, levodopa, major depressive disorder, parkinsonism

## Abstract

Geriatric depression often presents with psychomotor retardation. Severe psychomotor retardation may manifest as pseudoparkinsonism, which mimics Parkinson's disease (PD) and is often difficult to treat with standard antidepressant therapy. We report a case of a woman in her 60s with recurrent major depression with melancholic features who remained symptomatic despite several months of vortioxetine augmented with aripiprazole. She developed overt parkinsonism, including asymmetric rest tremor, rigidity, bradykinesia, and postural instability, requiring wheelchair assistance (Hoehn and Yahr stage 5). A diagnostic trial of levodopa produced marked improvement in both motor and depressive symptoms, leading to a presumptive diagnosis of comorbid PD. However, a subsequent dopamine transporter scan (DaTscan) showed normal striatal uptake, which argued against neurodegeneration. The final diagnosis was considered most consistent with geriatric depression with pseudoparkinsonism. During the 3 years of follow‐up, her depressive symptoms showed a clear temporal association with levodopa: symptoms worsened after discontinuation and remitted after reintroduction. This case highlights that pseudoparkinsonism in geriatric depression can closely mimic PD and that DaTscan may help exclude neurodegenerative parkinsonism. These findings also suggest that levodopa may be considered in selected cases of difficult‐to‐treat geriatric depression with pseudoparkinsonism.

## Key Clinical Message

Severe psychomotor retardation in geriatric depression may present as pseudoparkinsonism and closely mimic Parkinson's disease. Dopamine transporter imaging can assist in excluding neurodegenerative parkinsonism and avoiding misdiagnosis. Levodopa may be considered a therapeutic option in difficult‐to‐treat geriatric depression with pseudoparkinsonism.

## Graphical abstract

Long‐term clinical course of a patient with geriatric depression presenting with levodopa‐responsive pseudoparkinsonism over a 3‐year follow‐up period.

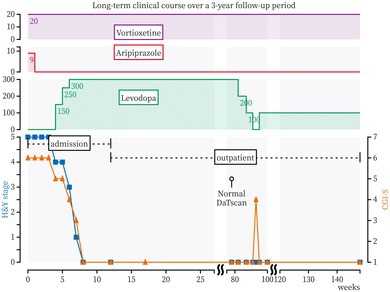

## 1 Introduction

Parkinson's disease (PD) is an age‐related neurodegenerative disorder, with incidence rates ranging from 108 to 212 cases per 100,000 persons aged ≥ 65 years, manifesting as asymmetric motor dysfunction, including bradykinesia, rest tremor, rigidity, and postural instability, which characterize primary parkinsonism [1]. Clinically, 38% of patients with PD experience depression as a non‐motor symptom [2], whereas individuals with depression have a twofold higher risk of developing PD [3].

Geriatric depression has a prevalence of approximately 10% among community‐dwelling adults aged ≥ 65 years [4]. According to the Diagnostic and Statistical Manual of Mental Disorders, Fifth Edition, Text Revision (DSM‐5‐TR), the features of major depressive disorder include depressed mood and anhedonia [5]. Patients may also present with weight loss, insomnia, fatigue, psychomotor agitation or retardation, inappropriate guilt, difficulty concentrating, and recurrent thoughts of death. Normal aging induces dopaminergic dysfunction driven by an increased inflammatory burden. This dysfunction leads to anhedonia and psychomotor retardation in geriatric patients with depression [6]. Psychomotor retardation involves both cognitive and motor slowing. Severe psychomotor retardation causes immobile facial expressions, bradyphrenia, stooped posture, and reduced gait speed. These clinical features mimic the motor symptoms of PD, including masked faces, rigidity, and bradykinesia. Clinicians define motor symptoms derived from psychomotor retardation as “pseudoparkinsonism.” This term distinguishes depressive motor dysfunction from primary parkinsonism observed in patients with PD [7].

Levodopa serves as a precursor to dopamine, crossing the blood–brain barrier and converting into dopamine within the brain. PD is characterized by a striatal dopamine deficiency. Levodopa improves motor symptoms by restoring dopamine deficiency [1]. Therefore, levodopa remains the first‐line treatment for patients with PD [8]. Furthermore, the clinical diagnostic criteria for PD include a dramatic improvement in motor symptoms after levodopa administration [9]. In contrast, evidence on the antidepressant effects of levodopa in patients with major depressive disorder is limited. The 2023 CANMAT guidelines for depression management do not include levodopa as an adjunctive medication for difficult‐to‐treat depression. Instead, dopamine‐related agents, such as dopamine stimulants or the dopamine agonist pramipexole, are listed as third‐line adjunctive options for difficult‐to‐treat depression [10]. Levodopa is not recommended even at this level.

We report the case of a woman in her 60s with recurrent geriatric depression who presented with overt parkinsonism during relapse of depression. Given the prominent motor symptoms and levodopa responsiveness, comorbid PD was suspected. However, a normal dopamine transporter scan (DaTscan) excluded neurodegeneration, leading to a final diagnosis of geriatric depression with pseudoparkinsonism. This case demonstrated that levodopa was associated with resolution of both motor and depressive symptoms. Over 3 years of follow‐up, depressive symptoms relapsed upon levodopa discontinuation and remitted upon reintroduction, suggesting a temporal relationship between levodopa treatment and depressive symptom changes.

## 2 Case History/Examination

The patient, a woman in her 60s and a former nurse, had no family history of mood disorders or PD. She had been psychiatrically well until approximately 16 years prior, when she experienced her first major depressive episode and achieved remission after 6 months of outpatient care. Ten years later, a second episode occurred following an earthquake and a traffic accident, requiring hospitalization. Three years thereafter, the third episode necessitated a 3‐month inpatient stay, where sertraline (100 mg) augmented with quetiapine (100 mg) led to marked improvement. One year later, a fourth episode was successfully treated with sertraline (100 mg) and aripiprazole (3 mg). After discharge, she maintained remission on maintenance therapy with sertraline and aripiprazole, participating in a daycare rehabilitation program and engaging in supported employment.

Two years after the fourth episode, despite ongoing maintenance therapy, she developed a fifth depressive episode, triggered by chronic pain from shoulder osteoarthritis and lumbar spondylosis that impaired her ability to work. Two months later, she was readmitted, at which time sertraline was replaced with vortioxetine (20 mg) while aripiprazole augmentation (6–9 mg) was maintained. Despite partial improvement and discharge after 1 month, her condition deteriorated the following month. Recurrent depressed mood, inappropriate guilt, and loss of motivation were accompanied by newly developed lower limb stiffness and gait instability. These combined psychiatric and motor symptoms markedly impaired her activities of daily living, rendering independent living at home impossible and necessitating the index admission.

## 3 Methods (Differential Diagnosis, Investigations, and Treatment)

Upon admission, psychiatric evaluation revealed a pervasive depressed mood, frequent tearfulness, and persistent guilt. She exhibited anhedonia, specifically, a loss of interest in her dogs. She also showed profound psychomotor retardation, with a low voice volume, prolonged response latency, and generalized slowness. In addition, she experienced insomnia, substantial weight loss, impaired concentration, and suicidal ideation. We ruled out manic episodes and the use of alcohol or depressogenic medications. Clinical laboratory tests revealed no evidence of thyroid dysfunction or systemic inflammation. Brain magnetic resonance imaging showed no brain atrophy or vascular lesions. Following the DSM‐5‐TR [5], we diagnosed her with recurrent major depressive disorder with melancholic features, specified by anhedonia, psychomotor retardation, excessive guilt, and anorexia. The Clinical Global Impression–Severity (CGI‐S) score was 6 [11].

Simultaneously, neurological examination revealed a masked face, marked bradykinesia, right‐hand rest tremor, left‐dominant rigidity, and postural instability, satisfying the Movement Disorder Society (MDS) clinical diagnostic criteria for parkinsonism [9]. Her daily living activities were severely impaired, requiring wheelchair use and assistance with transfers. Consequently, we suspected comorbid PD, with motor severity corresponding to Hoehn and Yahr (HY) stage 5 [12].

Vortioxetine (20 mg) was continued to treat depression. To exclude drug‐induced parkinsonism and validate the PD diagnosis, we discontinued aripiprazole and monitored the motor symptoms [9]. A diagnostic levodopa trial was planned if the parkinsonian motor symptoms persisted after aripiprazole withdrawal [9].

## 4 Conclusions and Results (Outcome and Follow‐Up)

### 4.1 Inpatient Management

**FIGURE 1:**
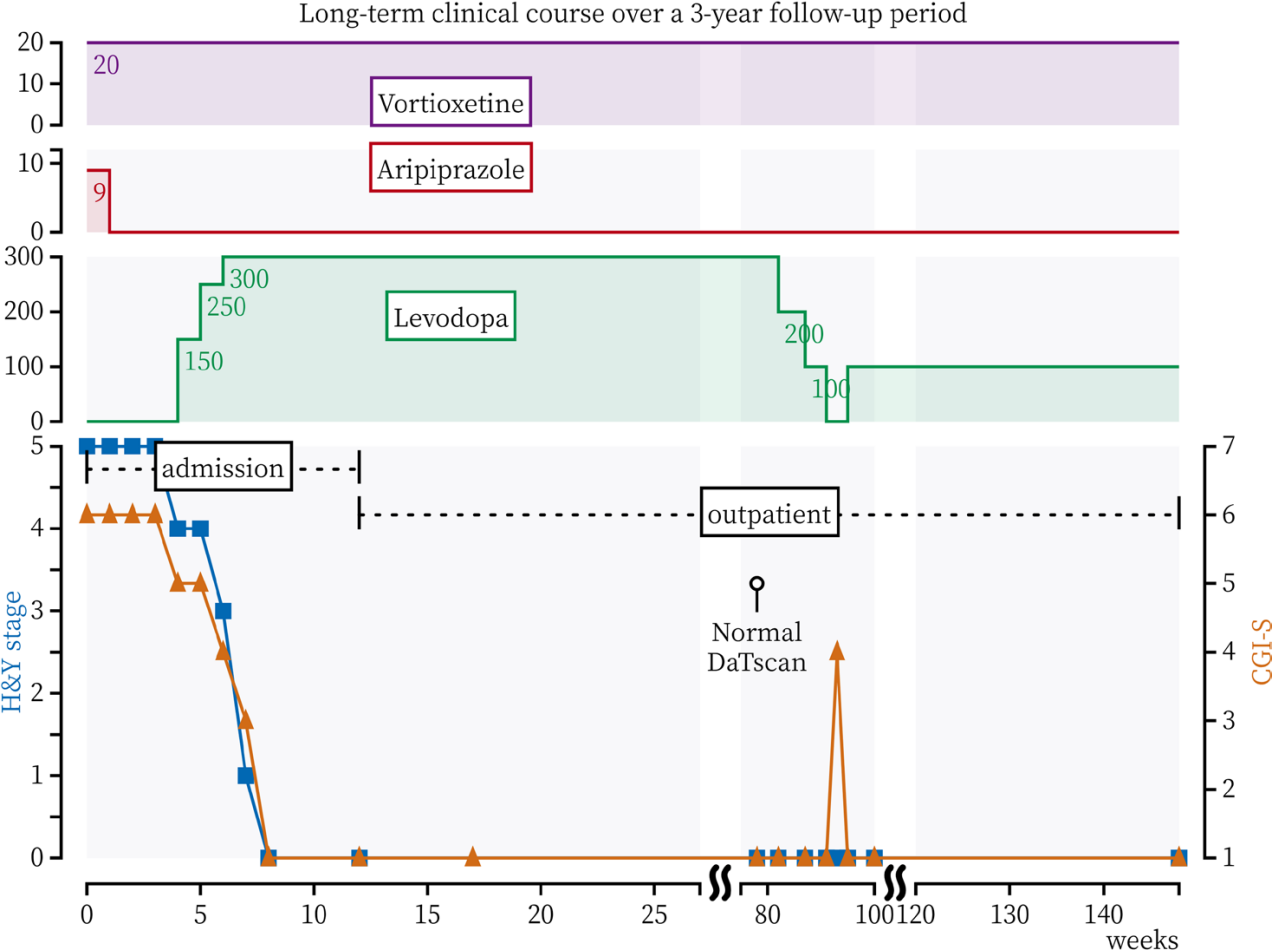
Long‐term clinical course over a 3‐year follow‐up period. Following admission and discontinuation of aripiprazole, treatment with levodopa (150–300 mg) was associated with marked improvement in parkinsonian motor symptoms (HY stage) and depressive symptoms (CGI‐S), enabling discharge at Week 12. A DaTscan at Week 78 demonstrated normal striatal uptake. Discontinuation of levodopa at Week 91 was followed by depressive relapse at Week 93, which improved after reintroduction of levodopa. Remission was sustained until Week 148 with vortioxetine and low‐dose levodopa (100 mg). CGI‐S, Clinical Global Impression–Severity scale; DaTscan, dopamine transporter scan; HY, Hoehn and Yahr stage.

By Day 5 of hospitalization, aripiprazole was discontinued to exclude drug‐induced parkinsonism. Despite a 2‐week washout period, severe motor dysfunction (HY stage 5) and depression (CGI‐S 6) persisted. At Week 4, a diagnostic trial of levodopa (150 mg) was initiated. She became able to rise from bed independently and ambulate short distances with a walker, although assistance was still required for most activities (HY stage 4). Response latency decreased, and appetite began to return (CGI‐S 5). By Week 6, the levodopa dose was raised to 300 mg/day. Bradykinesia and rigidity lessened, and gait speed improved. She became independent in activities of daily living but still exhibited mild postural instability (HY stage 3). Her motivation improved, and she began participating in group occupational therapy (CGI‐S 4). By Week 7, although mild unilateral rigidity remained, postural stability had normalized, and she was able to walk independently over long distances (HY stage 1). By Week 8, parkinsonian features had resolved completely (HY stage 0), and she was able to play table tennis. Both mood and pleasure had fully recovered (CGI‐S 1). Remission was sustained, and the patient was discharged at Week 12 (Figure 1).

As the severe motor symptoms persisted without improvement during the washout period after withdrawal of aripiprazole, we considered drug‐induced parkinsonism less likely, as it constitutes an absolute exclusion criterion in the MDS criteria. Dramatic motor recovery with levodopa and the presence of asymmetric rest tremor fulfilled two supportive criteria of the MDS [9]. Although rapid progression to wheelchair dependence was a red flag, the absence of absolute exclusions led to a diagnosis of clinically probable PD. Consequently, her discharge diagnosis was considered recurrent geriatric depression comorbid with PD, with dual follow‐up in the fields of psychiatry and neurology.

### 4.2 Outpatient Follow‐Up

After discharge, her symptoms remained stable with vortioxetine (20 mg) and levodopa (300 mg). Because the patient had difficulty accepting the PD diagnosis, the neurologist conducted a diagnostic reassessment. DaTscan yielded normal results at Week 78. This met an absolute exclusion criterion for the MDS, prompting the neurologist to withdraw the PD diagnosis and discontinue levodopa therapy. Levodopa was tapered and discontinued at Week 91. By Week 93, moderate depressive symptoms recurred (CGI‐S 4) without the return of overt parkinsonism. Upon reintroduction of levodopa (100 mg), her depressive symptoms remitted by Week 95. The final diagnosis was clinically interpreted as geriatric depression with severe psychomotor retardation, manifesting as pseudoparkinsonism. On a maintenance regimen of vortioxetine (20 mg) and levodopa (100 mg), the patient remained in remission for more than 1 year (Figure 1).

## 5 Discussion

The diagnosis of pseudoparkinsonism requires the systematic consideration of three alternative etiologies of parkinsonism. On admission, the patient presented with depressive symptoms and overt parkinsonism. The initial clinical course, which included the exclusion of drug‐induced causes, dramatic response to levodopa, and the presence of asymmetric rest tremor, led to a presumptive diagnosis of clinically probable PD. However, a normal DaTscan at Week 78 refuted the diagnosis of PD. The final diagnosis was revised to pseudoparkinsonism secondary to geriatric depression. Pseudoparkinsonism refers to the phenotype in which psychomotor retardation‐associated motor slowing in depression becomes symptomatically indistinguishable from parkinsonism [7]. Three differential diagnoses warrant systematic consideration. First, drug‐induced parkinsonism (DIP) cannot be completely excluded, particularly given the prior use of aripiprazole. Although aripiprazole carries a lower risk of DIP than full D2 antagonists owing to its partial D2 agonist profile, it remains a recognized causative agent, and parkinsonism may persist for weeks to months following withdrawal, particularly in elderly patients with a reduced dopaminergic reserve [13]. The 2‐week washout period in this case was therefore insufficient to confidently exclude DIP. However, two clinical observations suggest that a purely drug‐induced etiology is less likely. On the one hand, severe motor symptoms persisted without improvement during the 2‐week washout period after discontinuation of aripiprazole, whereas DIP typically shows at least partial improvement after withdrawal of the offending agent. On the other hand, the motor symptoms showed a clear dose‐dependent response to levodopa, a pattern not consistently reported in DIP [13]. Second, very early PD remains a theoretical possibility, despite normal DaTscan results [14]. Nevertheless, the absence of progressive motor deterioration over approximately 18 months following the normal DaTscan is inconsistent with the expected clinical course of PD. Third, functional parkinsonism (FP), a manifestation of functional neurological disorders, in most cases presents with highly variable and distractible motor symptoms and a lack of response to dopaminergic therapy [15]. The dose‐dependent response to levodopa observed in this case argues against the diagnosis of FP. After systematically evaluating these three alternatives, pseudoparkinsonism offers the most persuasive explanation, accounting for both the depressive and motor features within a single diagnostic framework of geriatric depression.

Two primary factors contributed to this diagnostic pitfall. First, the clinical presentation of her pseudoparkinsonism closely resembled the cardinal motor features of PD. A recent review noted that depressive pseudoparkinsonism typically lacks asymmetric rest tremor and fatigable bradykinesia, features that may aid in clinical differentiation from PD [7]. In our case, however, the unexpected presence of asymmetric rest tremor served as a major confounding factor, making differentiation from PD particularly challenging. Second, the patient's marked response to levodopa introduced a cognitive bias into the diagnostic process. Because levodopa is considered the gold standard treatment for PD and responsiveness to levodopa is regarded as a supportive diagnostic criterion [9], this therapeutic response may have reinforced an availability bias toward PD. This in turn contributed to premature closure, in which the apparent fulfillment of diagnostic criteria curtailed further systematic reconsideration of alternative explanations [16, 17]. The diagnosis was ultimately revised only after the collaborating neurologist prompted a reassessment of diagnostic certainty.

Dopamine transporter imaging can be a useful adjunct for excluding neurodegenerative parkinsonism by visualizing dopamine transporter availability in the nigrostriatal terminals [18]. Patients clinically diagnosed with PD but showing normal DaTscan results are categorized as having scans without evidence of dopaminergic deficits (SWEDDs), accounting for approximately 20% of such cases [19]. SWEDDs represent a heterogeneous group, including dystonia, essential tremor, and drug‐induced, vascular, or psychogenic parkinsonism. Notably, psychomotor retardation in depression is an important differential diagnosis in this spectrum [19]. Our case of geriatric depression with pseudoparkinsonism may also be considered part of the SWEDDs spectrum.

This case suggests that difficult‐to‐treat geriatric depression with pseudoparkinsonism may respond to levodopa. Despite several months of treatment with vortioxetine augmented with aripiprazole, her geriatric depression progressed, with psychomotor retardation worsening to the level of pseudoparkinsonism. However, the introduction of levodopa was associated with marked improvement in both depressive and motor symptoms. Compared with three previous reports of geriatric depression with pseudoparkinsonism, this case suggests a potential antidepressant role of levodopa. First, a prior case report described a patient in their 60s in whom both depressive and parkinsonian symptoms improved through serotonergic and noradrenergic activation with imipramine [20]. Second, another case report described modest improvement in an elderly patient with difficult‐to‐treat depression and severe psychomotor retardation following treatment with pramipexole, a D2/D3 receptor agonist [21]. Finally, a case of geriatric depression with parkinsonism and a normal DaTscan described adjunctive levodopa as ineffective for 6 months; remission occurred only after intensive care unit treatment following a suicide attempt, leaving the relationship between levodopa and mood improvement unclear [22]. In contrast, our patient demonstrated responsiveness to the dopamine precursor levodopa, with symptoms worsening after discontinuation and improving after reintroduction.

In this patient with geriatric depression, dopaminergic therapy proved indispensable throughout both the acute and maintenance phases. Levodopa ameliorated the acute depressive episode accompanied by pseudoparkinsonism, and during the maintenance phase, its discontinuation precipitated depressive relapse while its reintroduction restored remission. However, DaTscan results were normal, confirming the absence of nigrostriatal neurodegeneration. This clinical course may suggest an underlying hypodopaminergic state without structural neurodegeneration. Three mechanisms may contribute to dopaminergic hypofunction in geriatric depression. First, normal aging progressively impairs dopaminergic signaling by reducing dopamine transporter (DAT) availability, vesicular monoamine transporter 2 binding, and D2‐like receptor density [6]. Second, age‐related neuroinflammation disrupts presynaptic dopamine release and reuptake [6]. Third, striatal DAT availability has been shown to be lower in patients with major depressive disorder than in healthy controls in a meta‐analysis of in vivo neuroimaging studies [23]. Reduced striatal dopaminergic tone has been suggested as the neurobiological correlate of psychomotor retardation in geriatric depression and is distinct from the nigrostriatal denervation pattern characteristic of PD [6]. In the present case, psychomotor retardation‐associated motor slowing became sufficiently severe to be symptomatically indistinguishable from parkinsonism. Future studies should investigate whether pseudoparkinsonism reflects a severe form of this hypodopaminergic state without the neuronal loss seen in PD.

The interpretation of this case has several limitations. First, we did not perform standardized quantitative assessments using the Hamilton Depression Rating Scale (HAMD) or the Unified Parkinson's Disease Rating Scale (UPDRS) [24, 25]. Second, as a single case report, the findings may not be generalizable to a broader population.

## 6 Take Home Message

Severe psychomotor retardation in geriatric depression can closely mimic Parkinsonian motor symptoms, presenting as pseudoparkinsonism. Dopamine transporter imaging may help exclude neurodegenerative parkinsonism. Levodopa may be considered a therapeutic option in difficult‐to‐treat geriatric depression with pseudoparkinsonism.

## Author Contributions

**Kenshiro Imaizumi:** writing – original draft. **Junichi Goto:** writing – original draft, writing – review and editing. **Kazuya Isoda:** writing – review and editing. **Tetsuya Watanabe:** writing – review and editing. **Yohei Nakamura:** writing – review and editing. **Suzuha Oyamada:** writing – review and editing. **Masashi Toda:** writing – review and editing. **Koki Hirazumi:** writing – review and editing. **Sachi Yamashiro:** writing – review and editing. **Yoshihiro Nabeshima:** writing – review and editing.

## Funding

The authors have nothing to report.

## Ethics Statement

This case report was reviewed and approved by the Institutional Review Board of Yuge Neuropsychiatric Hospital (approval no. 304). All procedures were conducted in accordance with the ethical standards of the institutional and national research committees and the Declaration of Helsinki.

## Consent

Written informed consent was obtained from the patient for the publication of this case report.

## Conflicts of Interest

The authors declare no conflicts of interest.

## Data Availability

Data sharing not applicable to this article as no datasets were generated or analysed during the current study.
